# An efficient intrathecal delivery of small interfering RNA to the spinal cord and peripheral neurons

**DOI:** 10.1186/1744-8069-1-29

**Published:** 2005-09-28

**Authors:** Miaw-Chyi Luo, Dong-Qin Zhang, Shou-Wu Ma, Yuan-Yuan Huang, Sam J Shuster, Frank Porreca, Josephine Lai

**Affiliations:** 1 Department of Pharmacology, The University of Arizona Health Sciences Center, Tucson, AZ 85724, USA; 2 Neuromics, Minneapolis, MN 55438, USA

**Keywords:** rat, antinociception, sensory neurons, spinal cord, knockdown, RNA interference

## Abstract

We have developed a highly effective method for *in vivo *gene silencing in the spinal cord and dorsal root ganglia (DRG) by a cationic lipid facilitated delivery of synthetic, small interfering RNA (siRNA). A siRNA to the delta opioid receptor (DOR), or a mismatch RNA, was mixed with the transfection reagent, i-Fect™ (vehicle), and delivered as repeated daily bolus doses (0.5 μg to 4 μg) via implanted intrathecal catheter to the lumbar spinal cord of rats. Twenty-four hours after the last injection, rats were tested for antinociception by the DOR selective agonist, [D-Ala^2^, Glu^4^]deltorphin II (DELT), or the mu opioid receptor (MOR) selective agonist, [D-Ala^2^, N-Me-Phe^4^, Gly-ol^5^]enkephalin (DAMGO). Pretreatment with the siRNA, but not the mismatch RNA or vehicle alone, blocked DELT antinociception dose-dependently. The latter was concomitant with a reduction in the spinal immunoreactivity and receptor density of DOR, and in DOR transcripts in the lumbar DRG and spinal dorsal horn. Neither siRNA nor mismatch RNA pretreatment altered spinal immunoreactivity of MOR or antinociception by spinal DAMGO, and had no effect on the baseline thermal nociceptive threshold. The inhibition of function and expression of DOR by siRNA was reversed by 72 hr after the last RNA injection. The uptake of fluorescence-tagged siRNA was detected in both DRG and spinal cord. The low effective dose of siRNA/i-Fect™ complex reflects an efficient delivery of the siRNA to peripheral and spinal neurons, produced no behavioral signs of toxicity. This delivery method may be optimized for other gene targets.

## Background

RNA interference (RNAi) is an endogenous mechanism of RNA dependent degradation of specific mRNA by a protein complex called the RNA induced silencing complex (RISC) [1]. This mechanism was first characterized in the nematode *Caenorhabditis elegans *as an intrinsic protective response against invaded viral RNA [2]. The sequence specific substrate selectivity of RISC is dictated by its complex formation with certain double stranded, small interfering RNA (siRNA) that, in the nematode, is generated from viral RNA by an enzyme complex called DICER [3]. Subsequently, it was demonstrated that RNAi could be achieved in cultured mammalian cells by transfecting them with chemically synthesized siRNA [4], or by plasmid generated siRNA [5], thus establishing a new technology for functional genomics as well as drug target validation. In vertebrate experimental models, RNAi has been successfully applied to target exogenous and endogenous gene expression in organ systems such as liver, spleen, kidney, lung, and pancreas via systemic delivery [6-10]. However, this novel technology is not readily amenable to targeting nervous system genes because siRNA does not easily cross the blood brain barrier, and its uptake by neurons *in vitro *is poor [11]. While chemical modification of siRNA may enhance the efficiency of uptake of these molecules in cultured neurons as demonstrated recently [12], only a few studies to date succeeded in knocking down target genes in the central nervous system (CNS) [13-16]. We proposed that the limited advance made in the latter could be improved by establishing a paradigm that efficiently delivers the siRNA to the CNS and can be optimized for a variety of targets.

We have previously shown that the delta opioid receptor (DOR) can be effectively knocked down *in vitro *[17] and *in vivo *[18,19] by antisense oligodeoxynucleotide (ODN) treatment. *In vivo*, an antisense ODN to the DOR, at a dose of 12.5 μg (1.6 nmol), given twice daily, produced a robust inhibition, by day 3, of antinociception of the DOR selective agonist, [D-Ala^2^, Glu^4^]deltorphin II (DELT) when given intracerebroventricularly [18] or intrathecally [19]. We thus postulate that the DOR is a suitable prototypic nervous system gene target for the purpose of optimizing *in vivo *RNAi by siRNA based on our prior experience with delivery and dosing of ODN, time course of the knockdown and turnover rate of the DOR. Another critical advantage is the well-established protocols and reagents for measuring the expression and function of DOR. We show here that a low dose of 2 μg of siRNA when mixed with a transfection agent, i-Fect™, and given once daily by the intrathecal route, effectively abolishes the function of DOR in the lumbar spinal cord. A selective reduction in the transcript and protein level of DOR in the lumbar dorsal root ganglia (DRG) and in the lumbar dorsal horn of the spinal cord suggests that the inhibitory effect of the siRNA on DOR is specific to its knockdown of DOR expression. The uptake of the siRNA by cells in the DRG and the spinal cord is consistent with its site of action. The effect of the siRNA is abolished by scrambling its sequence, or by discontinuing the siRNA treatment. The effective dose of the siRNA requires the use of i-Fect™, which facilitates the uptake of the siRNA into target tissues.

## Results

### Intrathecal administration of a siRNA to DOR blocked spinal DELT antinociception

**Figure 1:**
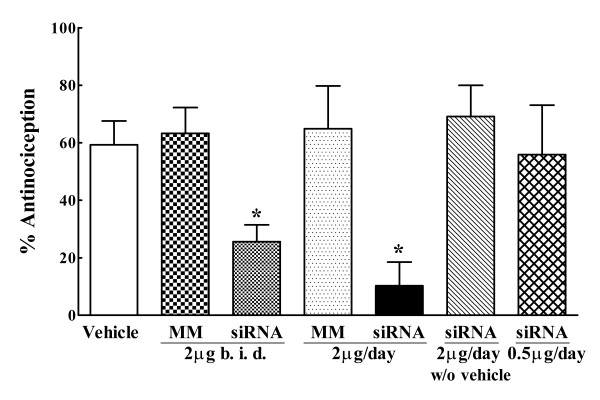
**Dose effect of intrathecal siRNA to DOR on the antinociceptive effect of the delta opioid agonist [D-Ala^2^, Glu^4^]deltrophin II (DELT). **DELT was given intrathecally (30 μg). The antinociceptive efficacy of DELT is defined as % antinociception measured 30 min after drug administration (n = 6 rats/group). Intrathecal DELT antinociception was significantly blocked after 3 consecutive once daily dose of siRNA at a daily dose of 4 μg (2 μg b.i.d.) or 2 μg (*p < 0.05), when compared with that observed in the vehicle control group. Mismatch RNA at the same doses had no effect on DELT antinociception. The 2 μg daily dose of siRNA, when administered without i-Fect™, did not block intrathecal DELT antinociception. The 0.5 μg dose of the siRNA in i-Fect™ did not have any effect on intrathecal DELT antinociception. Statistical analysis was carried out by one-way ANOVA following Dunnett's multiple comparisons.

The siRNA that was designed was first evaluated for its effect on DOR expression *in vitro*. This was accomplished by transfecting the siRNA/i-Fect™ complexes into NG108-15 cells that express endogenous DOR (the DOR in the NG108-15 cells has the mouse genotype; while the siRNA sequence was designed based on the rat DOR sequence, it cross-reacts with the mouse sequence). Quantitative RT-PCR analysis of total RNA extracts from these cells harvested 48 hr after transfection showed an 82.4 ± 9.2% knockdown of the DOR transcripts compared with that in control, non-transfected cells. For in vivo delivery, an initial experiment was conducted to determine the dose response of the siRNA, or mismatch RNA on intrathecal DELT antinociception (Fig. 1). Rats were given vehicle, the siRNA or mismatch RNA once daily for 3 consecutive days, and spinal DELT antinociception was measured 24 hr after the last injection. In the vehicle pretreated rats, intrathecal DELT produced 60 ± 8% MPE, which was consistent with that previously published [19]. The siRNA at 2 μg and 4 μg (as two 2 μg doses, one at 0900 and one at 1600 hr) daily dose both significantly attenuated the antinociception of DELT when compared with DELT antinociception in the vehicle control, while a 0.5 μg daily dose had no effect. The antinociception by DELT observed in the 2 μg and 4 μg siRNA pretreated groups were not significantly different from each other (10 ± 8% MPE, 25 ± 5% MPE, respectively; P > 0.05). The mismatch RNA treated groups did not differ significantly from the vehicle control irrespective of dosage of the mismatch RNA. These data established the 2 μg daily as the approximate minimum effective dose of this siRNA. However, if this dose of the siRNA was given without the vehicle i-Fect™, the subsequent antinociceptive effect of intrathecal DELT was robust, and was not significantly different from that observed in the vehicle pretreated group, or mismatch RNA treated groups (Fig. 1). Notably, none of the RNA treatments had any effect on the baseline thermal nociceptive threshold when compared with that of the vehicle control group. The baseline thermal latencies for paw withdrawal in the vehicle, siRNA (2 μg/day for 3 days), and mismatch RNA groups prior to treatment were: 20 ± 1.5 sec, 21 ± 1.1 sec, and 20 ± 1.1 sec, respectively. Twenty-four hr after the last injection, the baseline thermal latencies were 20 ± 1.0 sec, 19 ± 1.5 sec, and 20 ± 1.0 sec, respectively. Neither vehicle nor RNA treatment precipitated overt signs of behavioral toxicity or motor impairment in the animals. All subsequent siRNA or mismatch RNA treatment employed the 3 consecutive daily dose of 2 μg given with i-Fect™.

### The inhibitory effect of siRNA treatment on DELT antinociception is transient and reversible

**Figure 2:**
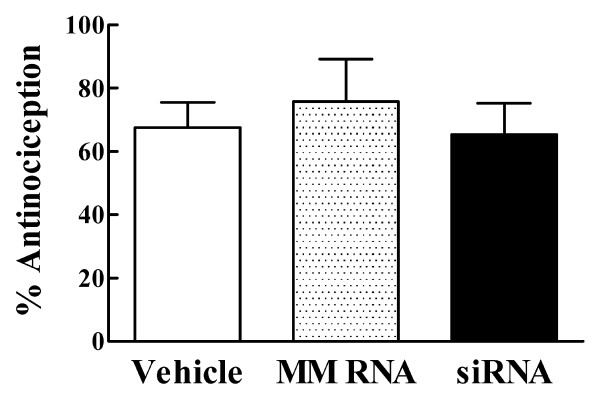
**Spinal DELT antinociception tested 72 hours after the last RNA or vehicle injection (n = 6/group). **There is no significant difference in the % antinociception among the three pretreatment groups (p > 0.05).

Whereas spinal DELT antinociception was attenuated after siRNA treatment, determined at 24 hr after the last siRNA injection, when the animals were tested again 48 hr later (i.e., 72 hr after the last injection), DELT antinociception was not different when compared with that in mismatch RNA and vehicle control groups (Fig. 2).

### Knockdown of DOR in the spinal dorsal horn by the siRNA

**Figure 3:**
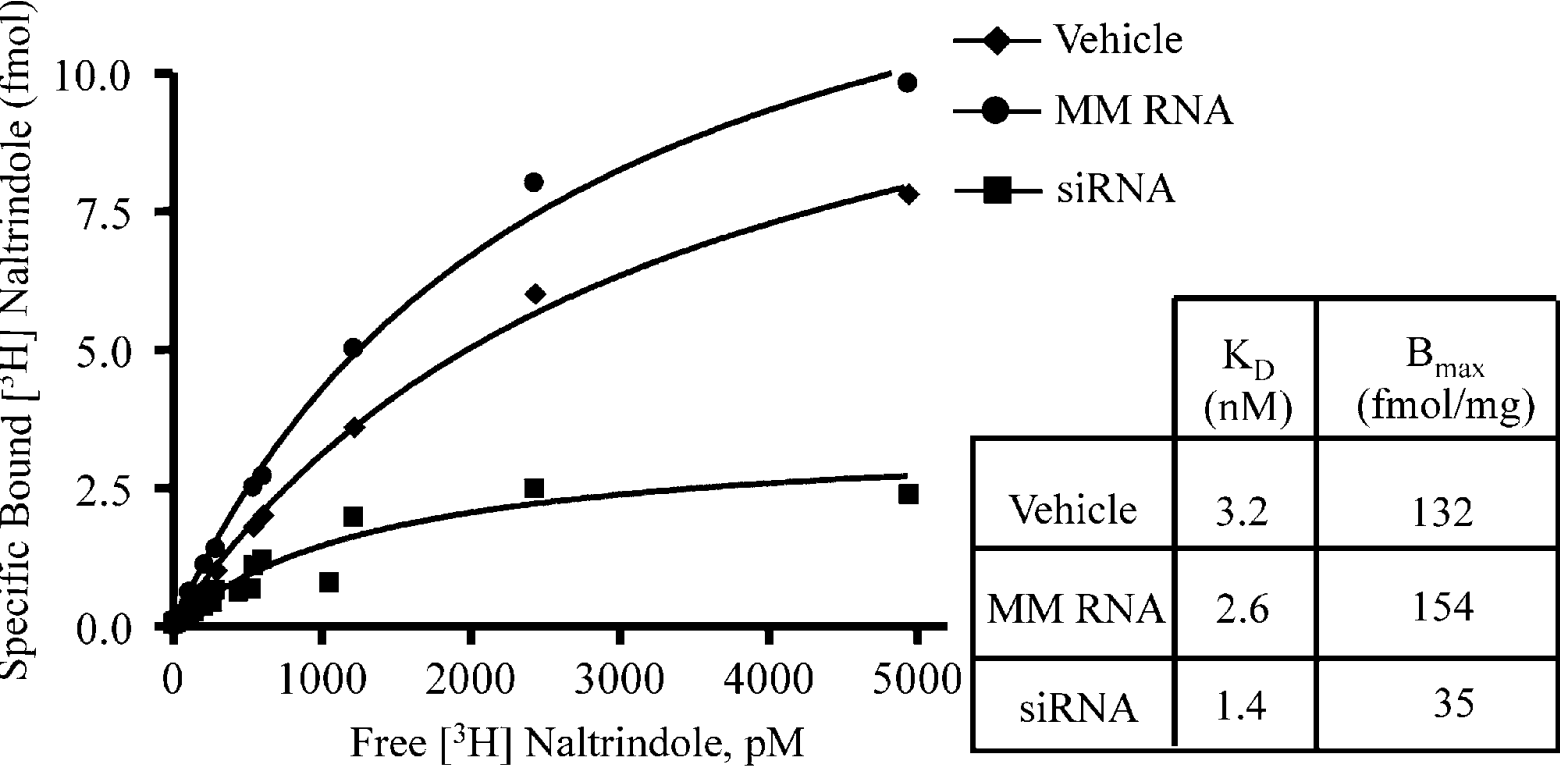
**Saturation [^3^H]naltrindole binding in membranes prepared from rat dorsal lumbar spinal cord (n = 3 rats/group). **The B_max _value in the siRNA group, but not the mismatch RNA group, was significantly lower than that in the vehicle treated group. The K_d _values are not significantly different among the 3 groups (p > 0.05). Data are representative of 3 independent experiments.

**Figure 4:**
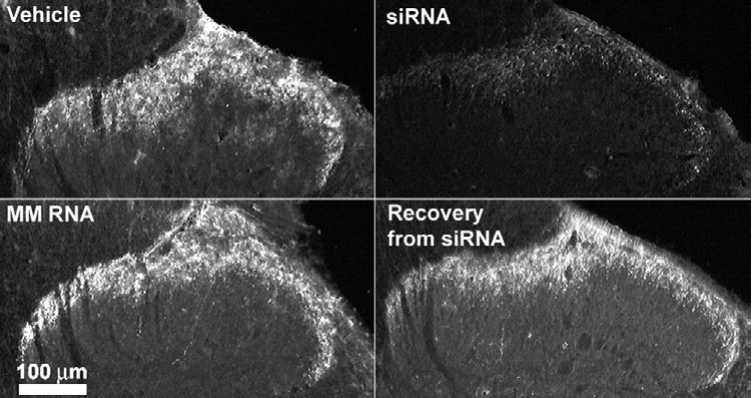
**DOR-immunoreactivity in the dorsal horn of the spinal cord after vehicle, siRNA or mismatch (MM) RNA treatment. **The immunoreactivity of DOR was predominantly found in laminae I/II of the dorsal horn. The immunolabeling was significantly lower in tissue from siRNA treated rats. Tissues taken 72 hours after the last siRNA injection (siRNA recovery) show similar level of immunoreactivity for DOR when compared with vehicle or MM RNA controls. Each image is representative of multiple sections from 3 rats per treatment group.

Figure 3 shows the saturation analysis of DOR in the spinal dorsal horn by the selective antagonist, [^3^H]naltrindole. SiRNA, but not mismatch RNA, produced a significant reduction in the density of the DOR measured as the maximum specific binding (B_max_) of the radioligand when compared with that in the vehicle treated controls. The reduction represents ~70% knockdown of the DOR density after siRNA treatment. The dissociation constant (K_d_) for [^3^H]naltrindole was not different in the three treatment groups. Immunohistochemical analysis using an anti-DOR antibody showed a significant reduction of immunoreactivity for DOR in the superficial laminae of the dorsal horn of the lumbar spinal cord from siRNA treated rats, but not from the mismatch RNA treated rats (Fig. 4). Lumbar spinal dorsal horn harvested from siRNA treated rats 72 hr after the last siRNA injection showed strong DOR immunoreactivity, which was not different from the vehicle or from the mismatch RNA controls. The recovery of DOR immunoreactivity by 72 hr after the last siRNA injection is consistent with the observed recovery of DELT antinociception in the siRNA treated rats at this time point shown in Fig. 2.

### siRNA treatment reduced the level of DOR transcripts in the DRG and the spinal dorsal horn

The mRNA levels of DOR in the lumbar spinal cord and L4/L5 DRGs were determined by quantitative RT-PCR at 24 hr after the last siRNA injection. In the siRNA treated group, there was a 38% reduction in DOR transcripts in the DRG (61.9 ± 5.7% of vehicle control), and 62% reduction in the lumbar spinal cord (38 ± 17.7% of vehicle control). Mismatch RNA pretreatment did not significantly alter the DOR mRNA level in either the DRG (109 ± 2.0% of vehicle control) or the lumbar spinal cord (90 ± 0.01% of vehicle control).

### siRNA was taken up by DRG and spinal cord cells

**Figure 5:**
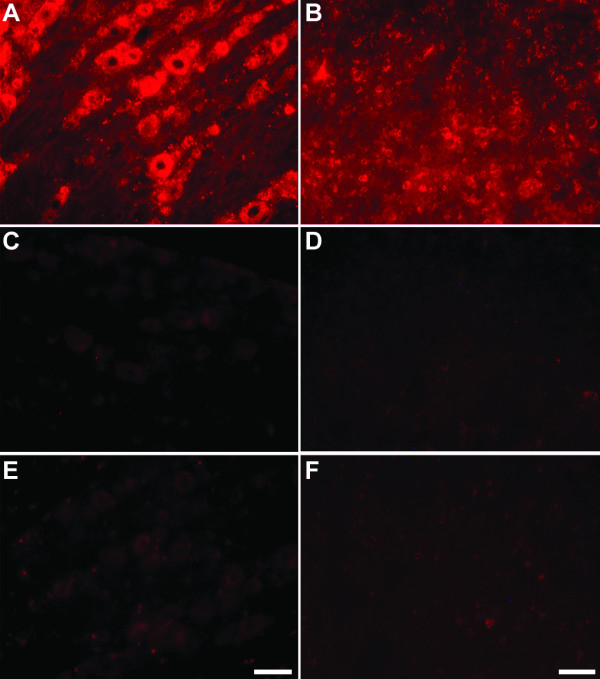
**AlexaFluor546-tagged siRNA uptake in lumbar DRG (A, C, E) and spinal cord (B, D, F). **Vehicle (10 μL) (C, D), siRNA with vehicle (2 μg/ 10 μL) (A, B), or siRNA without vehicle (2 μg/ 10 μL of aqueous annealing buffer) (E, F) was given intrathecally as a single injection. Tissues were harvested and processed 24 hr later. Images of the DRG were taken using a 20× objective lens and that of the spinal cord taken using a 40× objective lens. In all the fluorescence labeled cells, the labeling was punctate and peri-nuclear, and labeling intensities varied among cells. In the spinal cord (B), labeled cells were distributed widely in both the dorsal horn and the ventral horn. The images in B, D, F were taken from laminae I/II of the dorsal horn. Scale bar for the DRG images shown in panel E is 50 μm; scale bar for the spinal cord images shown in panel F is 25 μm.

AlexaFluor546-tagged siRNA fluorescence was clearly detected in both the lumbar DRG and spinal cord 24 hr after intrathecal delivery of 2 μg of the tagged RNA (Fig. 5A, B). In the DRG, fluorescence was present in cell bodies of various sizes. The labeling was punctate, perinuclear, and varied in intensity among the cells (Fig. 5A). In the spinal cord cross sections, fluorescence could be seen distributed over the entire cross sections, labeling a large number of cells in both the dorsal and the ventral horn, as well as the area around the central canal. Figure 5B shows an example of a 40 × magnification of the labeled spinal cord cells in the superficial laminae of the dorsal horn. The fluorescence is seen in the cytoplasm of the labeled cells, while nuclei typically appear as dark, unlabeled areas in the center of these cells. The localization of the fluorescence strongly suggests that the labeling is intracellular and thus due to the uptake of the tagged siRNA by the DRG and the spinal cord cells. Autofluorescence of the tissue sections was negligible under these imaging conditions when tissue sections from vehicle-injected rats were analyzed (Fig. 5C, D). Notably, when fluorescence-tagged siRNA was delivered into rats without i-Fect™, its uptake in the lumbar DRG (Fig. 5E) as well as in the lumbar spinal cord (Fig. 5F) was extremely poor.

### siRNA to DOR did not alter the function or expression of MOR

**Figure 6:**
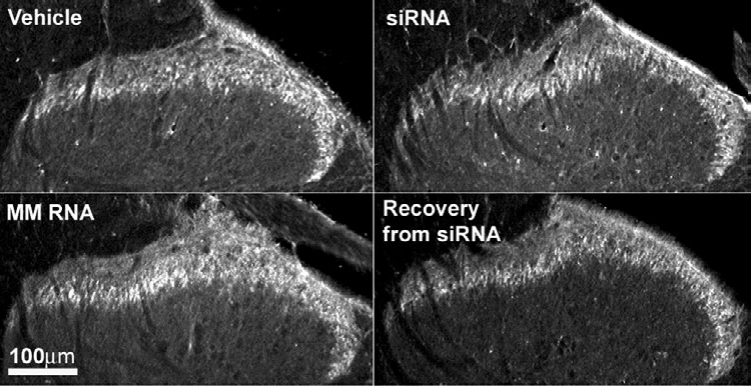
**MOR-immunoreactivity in the dorsal horn of the spinal cord after vehicle, siRNA (to DOR) or mismatch (MM) RNA treatment. **The MOR immunoreactivity is predominantly localized to laminae I/II of the dorsal horn. No significant difference in the immunoreactivity for MOR was seen among the 3 treatment groups. Tissue taken 72 hours after the last siRNA injection (siRNA recovery) also showed similar MOR immunoreactivity as the control groups. Each image is representative of multiple sections processed from 3 rats used in each group. The sections used in this figure and those used for DOR labeling shown in Figure 4 were from the same animals.

In vehicle-pretreated rats, DAMGO (0.5 μg) produced 66 ± 16% MPE, which is consistent with that previously published [20]. Neither siRNA to DOR, nor mismatch RNA treatment, had any effect on intrathecal DAMGO antinociception when compared with the vehicle control (79 ± 11% MPE, 73 ± 10% MPE, respectively to 66 ± 16% MPE; P > 0.05). There was also no significant difference in the MOR immunoreactivity in the dorsal horn of the spinal cord between siRNA, mismatch RNA, and vehicle treated groups (Fig. 6).

## Discussion

The advances made recently toward the *in vivo *applications of RNAi in vertebrate systems are critical towards developing siRNA as therapeutics [21-23]. These *in vivo *applications, however, do not yet apply to central nervous system function because siRNA do not readily cross the blood brain barrier (BBB) via systemic delivery. Because drugs may bypass the BBB by delivering them directly into the CSF, such delivery routes may be exploited in order to determine whether neurons and other cells in the nervous system may be amenable to RNAi by siRNA *in vivo*. Two recent reports used intrathecal delivery to prevent hypoxia-induced expression of brain derived neurotrophic factor in the spinal cord [14], or to knockdown the expression of the purinergic receptor subtype, P2X3, in sensory primary afferent [15]. While both studies demonstrated that siRNA attenuated the intended gene target, the former was a short intervention (3.5 hr) while the experimental subject was under general anesthesia, whereas the latter required very high concentration and continuous infusion of siRNA (400 μg/day for up to 7 days) to elicit a modest knockdown of the target. It is not clear whether the high dose of siRNA needed to effect reflects an inefficient RNAi mechanism in neurons or whether the modulation of gene function cannot be sustained over time.

Our data show that in the presence of a suitable transfection agent, the siRNA to the DOR is highly effective at a low concentration of 2 μg, or 0.14 nmol, given once a day. This dose is 23 times lower than the amount of antisense ODN required to elicit a knock down of the DOR as previously published [18], and is substantially lower than the effective dose reported for the knock down of the P2X3 receptors in the DRG [15]. The siRNA treatment delivered without the transfection reagent was without effect, suggesting that the transfection reagent significantly enhanced the uptake of the siRNA into target cells, as verified by the detection of fluorescence uptake of spinal cord and DRG tissues after injecting the tagged siRNA. This observation is consistent with recent evidence that the uptake of siRNA by neurons in culture is likely a key limiting step in the siRNA mediated gene silencing [12]. The amount of RNA delivered as a bolus dose in transfection reagent tends to be limited by the solubility of the RNA. The transfection reagent used in the present study was i-Fect™, which is a cationic lipid mixture that has been optimized for the delivery of short oligonucleotides in vitro [24]. This reagent was chosen for the present study because the RNA/lipid complexes remain in suspension at a fairly high concentration in a volume that is suitable for intrathecal delivery. A maximum of 2 μg of RNA can be given in a 10 μL injection volume. Should a daily dose of >2 μg is desired, the delivery can be adjusted by giving multiple doses.

A significant knockdown of DOR transcripts by siRNA treatment is consistent with the proposed mechanism of action of RNAi [25,26]. In this regard, the knockdown of the DOR transcripts was observed in both the dorsal horn of the spinal cord as well as the lumbar DRG, demonstrating that the siRNA interferes with the synthesis of both the presynaptic and the postsynaptic populations of DOR, which is also highly consistent with the uptake pattern of the tagged siRNA. The effects of siRNA culminate in a significant reduction of DOR immunoreactivity and ligand binding capacity in the superficial laminae of the dorsal horn of the spinal cord where the functional receptors are predominantly located. The loss of functional DOR is evident by the loss of antinociceptive activity of the DOR selective agonist, DELT, given intrathecally. Together, these results justify our conclusion that we have established an effective method for delivering siRNA that interferes efficiently with the expression and function of target genes in both the peripheral nervous system (i.e., sensory primary afferent) and the central nervous system (i.e., spinal cord).

The use of a mismatch RNA confirms the specificity of the siRNA sequence for the DOR. The siRNA treatment had no effect on the expression or the function of the highly homologous receptor type, MOR, further supporting the target specificity of the siRNA employed here. Finally, the effect of the siRNA is fully reversible; thus the observed effects of the siRNA are specific to the use of siRNA, and the treatment paradigm does not precipitate any long-term effects due to toxicity such as motor impairment. This paradigm can be easily adjusted for dosage and duration of treatment, and is based on a well-established, relatively non-invasive method of drug delivery that has general applications for spinal and peripheral targets. The reagents required are minimal and economical, and can be adapted for other gene targets. Our findings support the hypothesis that siRNA can be effectively applied to modulate nervous system function. The significant knockdown of the target transcripts in both the DRG and the spinal cord conforms with the established mechanism of siRNA mediated gene silencing, and is consistent with the uptake of siRNA seen in both the peripheral neurons (i.e. DRG) and the central nervous system (i.e., spinal cord). The low dose of siRNA further suggests that the efficacy of RNAi depends critically on the efficient delivery of the siRNA to the target tissues. Unlike antisense oligodeoxynucleotides, siRNA may be delivered systemically [23]. Thus, chemical modifications that enhance systemic stability and facilitate siRNA transport across the BBB or the uptake of siRNA by neurons would greatly advance the potential of siRNA as therapeutic.

## Methods

### siRNA preparation

The siRNA sequence for the DOR (accession no. U00475, the only gene sequence that is defined as delta opioid receptor to date) was from nucleotides 364 to 384 relative to the start codon. The sequences were as follows: sense 5'-GGCUGUGCUCUCCAUUGACUU-3'; antisense 5'-GUCAAUGGAGAGCACAGCCUU-3'. A scrambled sequence was designed as a mismatch control: sense 5'-GGCGUGUCUCUCUUACGACUU-3' and antisense 5'-GUCGUAAGAGAGACACGCCUU-3'. BLAST search of the nucleotide sequences in the GenBank database showed no substantial homology with other genes. These 21-nucleotide RNA oligonucleotides were synthesized individually, deprotected and purified by RNase-free HPLC (Midland Certified Reagent Company). siRNA and mismatch RNA duplexes were formed in a concentration of 200 μM in annealing buffer as described [4] for 3 min at 90°C followed by 1 h at 37°C. The siRNA stocks were aliquoted and stored at -80°C. For fluorescence labeling of the siRNA, AlexaFluor 546 was conjugated at the 5' end of the sense strand by solid phase synthesis and the tagged siRNA was purified by HPLC (Qiagen) and resuspended to a concentration of 200 μM in annealing buffer.

### In vitro analysis of knock down of DOR in NG108-15 cells by the siRNA

NG108-15 Cells were cultured in 5% fetal calf serum/ 5% newborn calf serum/ 45% Ham's F-12/ 45% DMEM/ 100 U mL^-1 ^penicillin/ 100 μg mL^-1 ^streptomycin (Invitrogen). Cells were maintained in 75 cm^2 ^flasks in a humidified atmosphere with 95% air and 5% CO2. For experiments, cells were seeded in 6-well plates at 3 × 10^5 ^cells/well 24 hr before the siRNA treatment. Cells were transfected with 2 μg of siRNA/i-Fect™ complexes in 1:4 ratio (w/v) per well. Forty-eight hours after transfection, cells were harvested and total RNA was isolated. DOR transcripts were detected by real-time RT-PCR (see below).

### Quantitative RT-PCR

Total RNA was isolated from the lumbar dorsal spinal cord, the L4 and L5 dorsal root ganglia using Aurum™ total RNA mini kit (Bio-Rad). Quantitative RT-PCR was performed using the iCycler iQ Multicolor Real-Time PCR Detection System with iScript cDNA Synthesis Kit and iQ SYBR Green Supermix (Bio-Rad). All samples were run in triplicate using an annealing temperature of 60°C. Primers for the amplification of DOR were: forward primer: 5'-GTTCACCAGCATCTTCACG -3'(nuc. 396~414); reverse primer: 5'-TGCATACCACTGCTCCATC -3'(nuc. 577~595). That for GAPDH were: forward primer: 5'-ATCATCCCTGCATCCACTG-3'(nuc. 610~628); reverse primer: 5'-GCCTGCTTCACCACCTTC -3'(nuc. 771–788). All primers were synthesized by Midland Certified Reagent Company, Inc. PCR efficiencies for DOR and GAPDH were 97% and 98%, respectively, with correlation coefficient of 0.999. Expression of DOR was normalized to expression of GAPDH. The differences of DOR mRNA expression between treatments were analyzed using the Comparative C_T _Method (ABI Prism 7700 Sequence Detection System User Bulletin #2, p11–15, 2001). The threshold cycle (C_T_) is defined as the cycle at which the amount of amplified PCR product from the target cDNA reaches a fixed threshold. In each treatment, ΔC_T _= C_T _for the target, DOR – C_T _for the endogenous reference, GAPDH. ΔΔC_T _= ΔC_T,treatment _– ΔC_T,control_. The equation, 2^-ΔΔCT^, denotes the ratio of the level of DOR transcripts in the treated group and that of the control group. This number is converted to percent of control, where control is set at 100.

### Animal surgery and RNA administration

Male Sprague-Dawley rats, weighing between 200–220 g, were used in these experiments. All the procedures used in these experiments have been approved by the Institutional Animal Care and Use Committee. Rats were implanted with intrathecal (*i.th.*) catheters and allowed 7 days to recover from surgery prior to treatment. The rats were divided into siRNA, mismatch RNA and vehicle groups, with at least 6 rats per group. siRNA or mismatch RNA complexes were prepared immediately prior to administration by mixing the RNA solution (200 μM in annealing buffer) with a transfection reagent, i-Fect™ (Neuromics), in a ratio of 1:4 (w:v) [24]. At this ratio, the final concentration of RNA as an RNA/lipid complex was 2 μg in 10 μL. siRNA or mismatch RNA, or i-Fect™ alone (defined as vehicle) in 10 μL was delivered to the lumbar region of the spinal cord via the *i.th. *catheters. Injections were given daily for 3 consecutive days. Nociceptive testing and tissue harvest were carried out at 24 hr and 72 hr after the last injection. The fluorescence tagged siRNA was mixed with i-Fect™ and injected in the same manner as for the untagged RNA, except that only one injection was given and tissue harvested 24 hr later.

### Nociceptive testing

Radiant heat paw withdrawal test using a movable infrared light source was employed as the nociceptive stimulus. The experimenter who conducted the nociceptive testing was blinded to the pretreatment of the experimental groups. The baseline latency for withdrawal of the left hindpaw was recorded from the experimental animals 24 hr prior to RNA or vehicle administration. Twenty-four hr following the last injection, the baseline latency was recorded again. The rats then received either 30 μg of [D-Ala^2^]deltorphin II (DELT) or 0.5 μg of [D-Ala^2^, N-Me-Phe^4^, Gly-ol^5^]enkephalin (DAMGO) (Sigma) intrathecally and the thermal latency were measured at 15-min interval over 60 min. A maximal cutoff latency of 33 s was used to prevent potential tissue injury. The % antinociceptive effect of DELT or DAMGO was defined as: [(treatment-baseline)/(cutoff-baseline)] × 100. The testing was repeated at 72 hr after the last injection.

### Saturation analysis of DOR by [^3^H]naltrindole

Crude membranes were prepared from the lumbar dorsal spinal cords pooled from the rats in each treatment group, resuspended in 50 mM Tris buffer, pH7.2, and the protein content measured as previously described [18]. Saturation analysis of [^3^H]naltrindole (20 Ci/mmol, PerkinElmer) was carried out using 10 concentrations of [^3^H]naltrindole (31.3 pM to 5 nM), done in triplicates, incubated with 50 μg of membranes per assay tube in 0.5 mL buffer, at a total volume of 1.0 mL, in a shaking water bath at 25°C for 3 hr. Non-specific binding of the radioligands was defined by that in the presence of 10 μM naloxone. The reactions were terminated by rapid filtration through Whatman GF/B filters, followed by five washes with 4 mL of ice-cold saline. The radioactivity was determined by liquid scintillation counting. Data were analyzed by non-linear least squares regression analysis using GraphPad Prism 4 (GraphPad software).

### Immunohistochemical analysis of DOR and MOR and fluorescence imaging

Rats were deeply anesthetized with ketamine HCl/xylazine (Sigma). The heart was exposed and transcardially perfused with 10 mM sodium phosphate-buffered saline (PBS, pH 7.4) until exudate ran clear, then switched to 10% buffered formalin for a further 15 min. Lumbar spinal cords and the L4 and L5 DRG were isolated and post-fixed in 10% buffered formalin for 24 hr and cryoprotected with 30% sucrose in 10 mM PBS. After pre-blocking with 10% normal goat serum (Vector Laboratories), frontal sections (20 μm) of the spinal cord were incubated with either a rabbit anti-rat primary antibody for DOR (1:2000) or a rabbit anti-rat primary antibody for MOR (1:5000) at 4°C for 48 hr (both antibodies were from Neuromics). Slides were rinsed with 2.5% normal goat serum/PBS and then incubated with a secondary antibody (AlexaFluor 594-conjugated goat anti-rabbit IgG (1:1000, Molecular Probes) for 1 hour at room temperature. Slides were again rinsed 3 times with the same buffer, dried, and mounted for microscopy. Fluorescent imaging of the samples was carried out using Nikon E800 fluorescence microscope equipped with 10×, 20× and 40×objective lenses and standard filters for Y-2E/C TX RED, coupled to a Hamamatsu C5810 color CCD camera (Hamamatsu Photonic System) for digital image acquisition using Adobe Photoshop software on a PC workstation. The same system was used for the imaging of slide mounted lumbar spinal cord and L4/L5 DRG (20 μm longitudinal sections) from animals that have been injected with the tagged siRNA, except that the filters for TRITC was used.

### Statistical analysis

Significant differences among paw withdrawal latencies at 30-min time point after DELT or DAMGO injection were determined by ANOVA followed by the *post hoc *least significant differences test. Unpaired t-test was used for all other between group comparisons.

## Abbreviations

RNAi RNA interference

siRNA Small interfering RNA

MM RNA Mismatch RNA

*i.th. *Intrathecal

DOR Delta opioid receptor

DELT [D-Ala^2^]deltrophin II

MOR Mu opioid receptor

DAMGO [D-Ala^2^, N-Me-Phe^4^, Gly-ol^5^]enkephalin

DRG Dorsal root ganglia

## Competing interests

The author(s) declare that they have no competing interests.

## References

[B1] Hammond SM, Bernstein E, Beach D, Hannon GJ (2000). A genetic link between co-suppression and RNA interference in *C. elegans*. Nature.

[B2] Fire A, Xu S, Montgomery MK, Kostas SA, Driver SE, Mello CC (1998). Potent and specific genetic interference by double-stranded RNA in *Caenorhabditis elegans*. Nature.

[B3] Bernstein E, Caudy AA, Hammond SM, Hannon GJ (2001). Role for a bidentate ribonuclease in the initiation step of RNA interference. Nature.

[B4] Elbashir SM, Harborth J, Lendeckel W, Yalcin A, Weber K, Tuschl T (2001). Duplexes of 21-nucleotide RNAs mediate RNA interference in cultured mammalian cells. Nature.

[B5] Brummelkamp TR, Bernards R, Agami R (2002). A system for stable expression of short interfering RNAs in mammalian cells. Science.

[B6] McCaffrey AP, Meuse L, Pham T-TT, Conklin DS, Hannon GJ, Kay MA (2002). Gene expression: RNA interference in adult mice. Nature.

[B7] Song E, Lee S-K, Wang J, Ince N, Quyang N, Min J, Chen J, Shankar P, Lieberman J (2003). RNA interference targeting Fas protects mice from fulminant hepatitis. Nat Med.

[B8] Sorensen DR, Leirdal M, Sioud M (2003). Gene silencing by systemic delivery of synthetic siRNAs in adult mice. J Mol Biol.

[B9] Zender L, Hutker S, Liedtke C, Tillmann HL, Zender S, Mundt B, Waltemathe M, Gosling T, Flemming P, Malek NP, Trautwein C, Manns MP, Kühnel F, Kubicka S (2003). Caspase 8 small interfering RNA prevents acute liver failure in mice. Proc Natl Acad Sci USA.

[B10] Kobayashi N, Matsui Y, Kawase A, Hirata K, Miyagishi M, Taira K, Nishikawa M, Takakura Y (2004). Vector-based in vivo RNA interference: dose- and time-dependent suppression of transgene expression. J Pharmaco Exp Ther.

[B11] Trülzsch B, Wood M (2004). Application of nucleic acid technology in the CNS. J Neurochem.

[B12] Davidson TJ, Harel S, Arboleda VA, Prunell GF, Shelanski ML, Greene LA, Troy CM (2004). Highly efficient small interfering RNA delivery to primary mammalian neurons induces microRNA-like effects before mRNA degradation. J Neurosci.

[B13] Makimura H, Mizuno TM, Mastaitis JW, Agami R, Mobbs CV (2002). Reducing hypothalamic AGRP by RNA interference increases metabolic rate and decreases body weight without influencing food intake. BMC Neurosci.

[B14] Baker-Herman TL, Fuller DD, Bavis RW, Zabka AG, Golder FJ, Doperalski NJ, Johnson RA, Watters JJ, Mitchell GS (2003). BDNF is necessary and sufficient for spinal respiratory plasticity following intermittent hypoxia. Nat Neurosci.

[B15] Dorn G, Patel S, Wotherspoon G, Hemmings-Mieszczak M, Barclay J, Natt FJC, Martin P, Bevan S, Fox A, Ganju P, Wishart W, Hall Jonathan (2004). siRNA relieves chronic neuropathic pain. Nucleic Acids Res.

[B16] Thakker DR, Natt F, Husken D, Maier R, Muller M, van der Putten H, Hoyer D, Cryan JF (2004). Neurochemical and behavioral consequences of widespread gene knockdown in the adult mouse brain by using nonviral RNA interference. Proc Natl Acad Sci.

[B17] Lai J, Crook TJ, Payne A, Lynch RM, Porreca F (1997). Antisense targeting of delta opioid receptors in NG108-15 cells: direct correlation between oligodeoxynucleotide uptake and receptor density. J Pharmacol Exp Ther.

[B18] Bilsky EJ, Bernstein RN, Hruby VJ, Rothman RB, Lai J, Porreca F (1996). Characterization of antinociception to opioid receptor selective agonists after antisense oligodeoxynucleotide-mediated "knock-down" of opioid receptor in vivo. J Pharmacol Exp Ther.

[B19] Bilsky EJ, Wang T, Lai J, Porreca F (1996). Selective blockade of peripheral delta opioid agonist induced antinociception by intrathecal administration of delta opioid receptor antisense oligodeoxynucleotide. Neurosci Lett.

[B20] Vanderah TW, Gardell LR, Burgess SE, Ibrahim M, Dogrul A, Zhong C-M, Zhang E-T, Malan TP, Ossipov MH, Lai J, Porreca F (2000). Dynorphin promotes abnormal pain and spinal opioid antinociceptive tolerance. J Neurosci.

[B21] Wood MJA, Trülzsch B, Abdelgany A, Beeson D (2003). Therapeutic gene silencing in the nervous system. Hum Mol Genet.

[B22] Rossi JJ (2004). A cholesterol connection in RNAi. Nature.

[B23] Soutschek J, Akinc A, Bramlage B, Charisse K, Constien R, Donoghue M, Elbashir S, Geick A, Hadwiger P, Harborth J, John M, Kesavan V, Lavine G, Pandey RK, Racie T, Rajeev KG, Röhl I, Toudjarska I, Wang G, Wuschko S, Bumcrot D, Koteliansky V, Limmer S, Manoharan M, Vornlocher H-P (2004). Therapeutic silencing of an endogenous gene by systemic administration of modified siRNAs. Nature.

[B24] More information about this reagent may be obtained on line at. http://www.neuromics.com.

[B25] Hannon GJ (2002). RNA interference. Nature.

[B26] Novina CD, Sharp PA (2004). The RNAi revolution. Nature.

